# Relationship of adhesion molecules (ICAM-1 and E-selectin) with ABO blood groups in patients hospitalized with acute myocardial infarction

**DOI:** 10.12669/pjms.40.7.8978

**Published:** 2024-08

**Authors:** Mohammad Perwaiz Iqbal, Iqbal Azam, Farzana Abubaker Yousuf, Khawar Kazmi

**Affiliations:** 1 Mohammad Perwaiz Iqbal, Department of Life Sciences, University of Management and Technology, Lahore, Pakistan. Pakistan Academy of Sciences, Islamabad, Pakistan. Department of Biological & Biomedical Sciences, Aga Khan University, Karachi, Pakistan; 2 Iqbal Azam, Department of Community Health Sciences, Aga Khan University, Karachi, Pakistan; 3 Farzana Abubaker Yousuf, Department of Biological & Biomedical Sciences, Aga Khan University, Karachi, Pakistan; 4 Khawar Kazmi, Department of Preventive Cardiology, National Institute of Cardiovascular Diseases, Karachi, Pakistan

**Keywords:** ABO blood groups, Adhesion molecules, ICAM-1, E-selectin, Acute myocardial infarction, Association study

## Abstract

**Objectives:**

Adhesion molecules, sICAM-1 and sE-selectin appear to have a major role in the pathogenesis of coronary artery disease (CAD). The focus of this study was to investigate the relationship of sICAM-1 and sE-selectin with ABO blood groups in Pakistani patients hospitalized with acute myocardial infarction (AMI).

**Methods:**

In a case-control study, 116 patients of acute myocardial infarction (AMI) and 116 healthy controls (age range for both: 30 years to 70 years; both males and females) were randomly selected from the Aga Khan University and National Institute of Cardiovascular Diseases, Karachi with informed consent. The blood samples were obtained and analyzed for ABO blood groups and serum levels of sICAM-1 and sE-selectin using kit methods. Statistical tests including independent sample t-test and Two-way ANOVA were used to study the association of these adhesion molecules with blood groups in AMI patients and healthy controls. Duration of the study was from July 2021 to June 30, 2023.

**Results:**

Mean serum levels of sICAM-1 were significantly higher in AMI patients compared to healthy controls (342±159 mg/dl vs. 227±104 mg/dl; p-value<0.001). Similarly, serum levels of sE-selectin were also significantly higher in AMI patients compared to healthy controls (53.6±26.9 mg/dl vs. 40.7± mg/dl; p-value<0.001). Moreover, mean concentrations of sICAM-1 and sE-selectin for the interaction between subject type (cases and control) and blood groups were statistically significant (p-value = 0.007 and p-value = 0.035, respectively).

**Conclusion:**

There is an association of adhesion molecules, sICAM-1 and sE-selectin with ABO blood groups in Pakistani patients hospitalized with AMI.

## INTRODUCTION

Coronary artery disease (CAD) is on the rise in Pakistan. According to recent data from WHO, 240,720 people in Pakistan died of coronary heart disease (CHD) in 2020.^1^ This has put Pakistan in the 30^th^ position in the world in terms of mortality due to CHD. A recent report has shown that there has been 10% rise in heart-related deaths since 2016.^2^ A meta-analysis by Zaman et al., indicated that South-Asian ethnicity is associated with higher incidence of CHD, compared to white Caucasions.^3^

The process of inflammation which is central to the development of atherosclerosis is facilitated by various adhesion molecules expressed on leukocytes and vascular endothelial cells.^4^-^6^ These adhesion molecules such as soluble intracellular adhesion molecule 1 (ICAM-1), soluble E-selectin, and soluble P-selectin have been shown to be biomarkers of inflammation, and increased serum levels of one or more of these molecules have been found to be associated with CHD.^7^ Kiechl et al. in a meta-analysis reported that circulating levels of sICAM-1, sP-selectin, and sE-selectin were associated with variations in the ABO locus.^8^ Larson et al. have shown an association of ABO blood groups with the adhesion molecules sICAM-1, sP-selectin, and sE-selectin in various ethnic groups in USA.^9^ Similarly, Zhang et al. have also reported an association of sICAM-1 and sP-selectin with ABO blood groups in a Chinese population.^7^

However, there are no reported studies carried out in Pakistan on the relationship between ABO blood groups and serum levels of adhesion molecules in normal healthy population as well as patients suffering from AMI. The present study was undertaken to investigate any relationship of ABO blood groups with sICAM-1 and sE-selectin in hospitalized AMI patients as well as healthy controls in Karachi.

## METHODS

In a case-control design, 116 AMI patients and 116 normal healthy subjects were randomly selected from the samples collected for the main study, entitled “Association of ABO genotypes with AMI in a Pakistani population” to investigate the association of adhesion molecules sICAM-1 and sE-selectin with ABO blood groups in hospitalized AMI patients and healthy controls with informed consent. The main study has been published in 2023.^10^ The sample size for the present nested study was estimated based on the estimates of BMI, WC, SBP, DBP, ICAM1 and E-selection in a published study.^7^ A maximum sample of 115 AMI patients and 115 normal healthy subjects was needed to achieve 80 percent power with the anticipated precision (mean difference) ranging between 2 (BMI) to 5 (SBP) between AMI patients and healthy controls, standard deviation among healthy individuals of 4.3 (BMI) to 15.0 (SBP), standard deviation among AMI patients of 3.9 (BMI) to 16.0 (SBP) and a two sided level of significance of 5.0%. The age range of participants was 30 years to 70 years and they included both males and females.

### Ethical Approval

It was obtained from Ethics Review Committees of both Aga Khan University [Ref: 3048-BBS-ERC-14] and National Institute of Cardiovascular Diseases (NICVD) [Ref: ERC-10/2015].

### Inclusion & Exclusion Criteria

The inclusion and exclusion criteria have been defined previously.^10^ Briefly, the AMI patients were recruited from the Aga Khan University Hospital and NICVD, Karachi. They were diagnosed to have AMI as per WHO guidelines for clinical practice which included high levels of troponin-I and electrocardiogram (ECG) changes. Though the sampling was from two different hospitals in Karachi, however, the proportions of male and female AMI patients were similar in the 2 hospitals. Normal healthy control subjects had been recruited from the personnel of these two hospitals. Their age range was between 30 years to 70 years, and they had no history of CAD.

Both patients and healthy controls had no history of liver disease, malabsorption syndrome, tuberculosis, uremia or cancer. Pregnant females and those using contraceptives were not included in the study. Waist circumference (WC) was obtained using a measuring tape at the mid-point between iliac crest and lower rib margin. Blood pressure was obtained using a sphygmomanometer. The duration of study was from July 2021 to June 2023.

### Blood sample collection and serological testing of adhesion molecules

From each subject, 10 ml of venous blood was obtained with informed consent. Four ml blood was transferred to a citrated tube for ABO blood group determination using the kit method (Antec Diagnostic Products, UK), while rest of the blood was transferred to a plain tube. After coagulation, the tubes were centrifuged at 1500*g* for 15 minutes to separate the serum, which was then aliquoted and stored at -20^o^C. These samples were later analyzed for determination of total cholesterol using a kit (Roche Diagnostics, USA), while concentration of sICAM-1 and sE-selectin were determined using the ELISA-based kits [Cat# DSLE00 for sICAM-1 and Cat# DCD540 for sE-selectin] obtained from RiboServ Inc. (Randolf, MA, USA) following instructions of the manufacturer.

### Statistical analysis

Demographic and clinical data were analyzed using SPSS version 19 for Windows. Means were expressed as mean±SD, while proportions of males and females were expressed as percentages. The percentage values were compared using chi-square test, while means were compared using independent sample t-test. For comparison of mean values of variables of different blood groups between normal healthy controls and AMI patients and interaction among subjects and blood groups, Two-way ANOVA was used. A p<0.05 was considered statistically significant.

## RESULTS

Demographic and clinical characteristics of the normal healthy controls and AMI patients have been presented in Table-I. There was a greater proportion of males as compared to females in these randomly selected subjects (p-value <0.001). Regarding the age and BMI, significant differences were observed between healthy subjects and AMI patients. However, no statistical difference was observed among patients and healthy controls with respect to waist circumference, blood pressure and total serum cholesterol. Mean serum concentrations of sICAM-1 and sE-selectin were significantly higher in AMI patients compared to normal healthy subjects (p-value <0.001). Distribution of frequencies of ABO blood groups among AMI patients and healthy controls is shown in Table-II. The percentages of B group were the highest in both cases and controls. For studying the association of two adhesion molecules with ABO blood groups, Two-way ANOVA was employed. Mean sICAM-1 concentrations were marginally significant between AMI patients and healthy controls (p-value = 0.08), whereas mean concentrations of sICAM-1 among different blood groups were similar (p-value = 0.539) (Table-III). On the other hand, mean sICAM-1 concentration values for the interaction between subject type (healthy controls and AMI patients) and blood groups were found to be highly significant (p-value = 0.007). Similarly, mean sE-selectin concentration values were also marginally significant between AMI patients and healthy controls (p-value = 0.091), while mean values of this adhesion molecule among the four blood groups were not significantly different (p-value = 0.118). However, mean sE-selectin values for the interaction between subject type and blood groups were statistically significant (p-value = 0.035).

**Table-I:** Demographic and clinical characteristics of the normal healthy controls and AMI patients in a Pakistani population.

Characteristics	Normal healthy controls (n =116) n (%)	AMI patients (n =116) n (%)	p-value*
Gender			
Males	83(71.6)	100(86.2)	< 0.001
Females	33 (28.4)	16 (13.8)	

	***Mean ± SD***	***Mean ± SD***	

Age (years)	41.6 ± 7.85	53.0 ± 8.9	< 0.001
BMI (kg/m^2^)	26.3 ± 3.7	24.5 ± 4.4	< 0.001
WC (cm)	91.8 ± 9.5	94.1 ± 9.4	0.07
SBP (mm Hg)	118 ± 12	116 ± 13	0.502
DBP(mm Hg)	78±8	76±10	0.106
ICAM1(mg/dl)	227±104.8	342.5±159.5	<0.001
E-selectin(mg/dl)	40.7±18.7	53.6±26.9	<0.001

BMI: body mass index, WC: waist circumference, SBP: systolic blood pressure, DBP: diastolic blood pressure.

* p-value represents the comparison of percentages in two groups using chi-square test, while the means were compared using independent sample t test. A p-value < 0.05 was considered as statistically significant.

**Table-II:** Distribution frequencies of ABO blood groups among AMI patients and healthy controls

Blood Group	AMI Patients (n=116)	Healthy Controls (n=116)

	n (%)	n (%)
A	30 (25.86)	27 (23.27)
B	46 (39.66)	45 (38.79)
O	28 (24.14)	34 (29.31)
AB	12 (10.34)	10 (8.62)

**Table-III:** Demographic and clinical characteristics of healthy controls and acute myocardial infarction (AMI) patients based on blood groups.

Healthy Controls					AMI Patients				p- value*		

Variables	AB Mean±SD	A Mean±SD	B Mean±SD	O Mean±SD	AB Mean±SD	A Mean±SD	B Mean±SD	O Mean±SD	Subject Type	Blood Group	Subject Type* Blood Group
Age (years)	41.1 ± 8.4	42.5 ± 8.8	41.4 ± 8.0	41.4 ± 7.1	50.3 ± 10.6	52.6 ± 9.4	52.9 ± 8.3	54.9 ± 8.7	<0.001	0.532	0.650
BMI (kg/m^2^)	27.1 ± 4.1	27.0 ±3.6	25.4 ± 3.7	26.7 ± 3.6	22.9 ± 4.7	23.7 ± 3.8	24.5 ± 4.8	25.9 ± 3.9	0.048	0.569	0.131
WC (cm)	93.4 ± 12.6	92.4 ± 10.2	89.5 ± 8.4	94.1 ± 9.1	88.8 ± 12.0	94.3 ± 9.8	94.4 ± 9.3	95.7 ± 7.7	0.608	0.518	0.189
SBP (mm of Hg)	118.5 ± 9.4	119.6 ± 9.9	118.9 ± 15.8	115.4 ± 8.7	112.5 ± 11.4	118.7 ± 13.3	117.5 ± 14.0	115.9 ± 11.6	0.173	0.207	0.784
DBP (mm of Hg)	77.5 ± 6.3	78.5 ± 7.6	80.0 ± 10.4	76.3 ± 6.1	73.3 ±7. 8	78.2 ± 12.0	76.9± 10.8	74.7 ± 8.8	0.049	0.118	0.781
***Biomarkers***											
TC (mg/dl)	180.6 ± 26.4	170.8 ± 29.6	178.6 ± 22.3	166.6 ± 27.4	158.4 ± 50.3	169.4 ± 34.8	159.0 ± 54.2	168.9 ± 32.1	0.198	0.992	0.202
ICAM-1 (mg/dl)	306.2 ± 176.7	214.8 ± 110.7	221.0 ± 71.4	221.6 ± 105.9	314.5 ± 97.2	258.6 ± 81.3	386.4 ± 205.3	372.4 ± 121.4	0.08	0.539	0.007
E-Selectin (mg/dl)	38.8 ± 11.4	29.0 ± 13.0	44.5 ± 17.9	45.4 ± 21.6	37.3 ± 26.2	41.3 ± 21.0	55.0 ± 26.9	71.6 ± 22.1	0.091	0.118	0.035

* p-value represents comparisons of means using Two Way ANOVA. A p-value < 0.05 was considered as statistically significant.

## DISCUSSION

The most significant finding of the present study was the association of adhesion molecules, sICAM-1 and sP-selectin with ABO blood groups in hospitalized AMI patients. Role of ABO blood groups with respect to their relationship with MI has been under investigation for a number of years. A couple of studies in South Asia have revealed an association of BB genotype with CAD indicating a high risk of AMI in B blood group individuals.^10^,^11^ This is in line with the previous research reports that non-O blood groups had higher risk of CAD compared to O blood group subjects.^12^ Moreover a meta-analysis of several studies showed that the relative risk of CHD was 1.11 (p-value = 0.001) in non-O blood group compared to O blood group.^13^ The intriguing question is about possible biochemical link between non-O blood group subjects and increased risk of developing atherosclerosis. A few research studies have shown that various soluble adhesion molecules are associated with development of atherosclerosis.^5^,^14^

sICAM-1 is a proinflammatory and proatherogenic cytokine which is associated with atherosclerosis and hence CAD.^15^ Similarly, sE-selectin is another inflammatory marker which is produced by damage to the endothelial cells and is associated with ABO blood groups.^16^ Therefore, these two adhesion molecules along with inflammation provide a potential link between ABO blood groups and pathogenesis of CAD.

In the univariate analysis, we found serum levels of both sICAM-1 and sE-selectin significantly increased in AMI patients compared to healthy controls. These findings corroborate previous results obtained by Ahmed et al. who have also shown significantly increased mean serum levels of sICAM-1 in CAD patients compared to normal controls (1593+85.3 ng/ml vs. 576+52.4 ng/ml). 17 Similar results have been reported in a study carried out in India in which sICAM-1 levels were significantly higher in CAD patients (>50% stenosis) compared to controls (<30% stenosis) with values 472.5+134.2 ng/ml vs. 410+142 ng/ml, respectively.^11^

Khoshbin et al. have reported significantly increased levels of sE-selectin, in CAD patients compared to controls in an Iranian population.^18^ As regards relationship of ABO blood group antigens with expression of sICAM-1 and sE-selectin, we did not find any significant difference in mean serum levels of these two adhesion molecules among healthy controls and AMI patients (Table-III). However, these findings are in conflict with the results reported by Zhang et al. indicating significantly decreased levels of sICAM-1 in A blood group of a healthy Chinese population and Larson et al. showing lower levels of sE-selectin in A_1_ allele subjects compared to O blood groups.^7^,^9^ This could be due to a relatively small sample size of healthy controls and AMI patients in the present study. Conversely, mean serum levels of sICAM-1 and sE-selectin for the interaction between subject type (controls and AMI patients) and blood groups were found to be statistically significant. Since Karachi is a multi-ethnic society, our recruited AMI patients and healthy controls represent four major ethnicities of Pakistan. This may be considered as a strength of this study.

### Limitations

One of the limitations of the present study is the unavailability of angiographic data about percentage stenosis in coronary vessels for several patients, there is evidence that mean serum levels of sICAM-1 are significantly different between single-vessel disease and triple-vessel disease.^17^ Despite this limitation, we have found association of these two adhesion molecules with ABO blood groups in Pakistani hospitalized AMI patients.

## CONCLUSION

In a population of hospitalized AMI patients, a significant association was found between soluble ICAM-1 and soluble E-selectin and ABO blood groups. Mean serum levels of these two adhesion molecules were significantly higher in AMI patients compared to healthy controls. The results suggest a possible link between type of blood group and expression of sICAM-1 and sE-selectin in the pathogenesis of CAD.

### Author’s Contribution:

**MPI** and **KK:** Conceived the Experiment.

**FAY, IA** and **MPI:** Were involved in data collection and statistical analysis and interpreted the results.

**MPI** and **IA:** Drafted the manuscript.

**MPI, KK** and **IA:** Reviewed the manuscript.

**FAY, MPI** and **IA:** Were responsible for the accuracy and integrity of work.
